# Role of matrix metalloprotease-2 and MMP-9 in experimental lung fibrosis in mice

**DOI:** 10.1186/s12931-022-02105-7

**Published:** 2022-07-08

**Authors:** Tina Bormann, Regina Maus, Jennifer Stolper, Meritxell Tort Tarrés, Christina Brandenberger, Dirk Wedekind, Danny Jonigk, Tobias Welte, Jack Gauldie, Martin Kolb, Ulrich A. Maus

**Affiliations:** 1 Division of Experimental Pneumology, Hannover Medical School, Feodor-Lynen-Strasse 21, 30625 Hannover, Germany; 2 Institute of Functional and Applied Anatomy, Hannover Medical School, Carl-Neuberg-Strasse 1, 30625 Hannover, Germany; 3 Institute of Laboratory Animal Science, Hannover Medical School, Carl-Neuberg-Strasse 1, 30625 Hannover, Germany; 4 Department of Pathology, Hannover Medical School, Carl-Neuberg-Strasse 1, 30625 Hannover, Germany; 5 Clinic for Pneumology, Hannover Medical School, Carl-Neuberg-Strasse 1, 30625 Hannover, Germany; 6 Department of Medicine, Pathology, and Molecular Medicine, McMaster University, 1280 Main St W, Hamilton, ON L8S 4L8 Canada; 7 German Center for Lung Research, Partner Site BREATH, Carl-Neuberg-Strasse 1, 30625 Hannover, Germany

**Keywords:** Gelatinase, IPF, Lung, Matrix metalloproteases

## Abstract

**Background:**

Idiopathic pulmonary fibrosis (IPF) is a diffuse parenchymal lung disease characterized by exuberant deposition of extracellular matrix (ECM) proteins in the lung interstitium, which contributes to substantial morbidity and mortality in IPF patients. Matrix metalloproteinases (MMPs) are a large family of zinc-dependent endopeptidases, many of which have been implicated in the regulation of ECM degradation in lung fibrosis. However, the roles of MMP-2 and -9 (also termed gelatinases A and B) have not yet been explored in lung fibrosis in detail.

**Methods:**

AdTGF-β1 was applied via orotracheal routes to the lungs of WT, MMP-2 KO, MMP-9 KO and MMP-2/-9 dKO mice on day 0 to induce lung fibrosis. Using hydroxyproline assay, FlexiVent based lung function measurement, histopathology, western blot and ELISA techniques, we analyzed MMP-2 and MMP-9 levels in BAL fluid and lung, collagen contents in lung and lung function in mice on day 14 and 21 post-treatment.

**Result:**

IPF lung homogenates exhibited significantly increased levels of MMP-2 and MMP-9, relative to disease controls. Enzymatically active MMP-2 and MMP-9 was increased in lungs of mice exposed to adenoviral TGF-β1, suggesting a role for these metalloproteinases in lung fibrogenesis. However, we found that neither MMP-2 or MMP-9 nor combined MMP-2/-9 deletion had any effect on experimental lung fibrosis in mice.

**Conclusion:**

Together, our data strongly suggest that both gelatinases MMP-2 and MMP-9 play only a subordinate role in experimental lung fibrosis in mice.

**Supplementary Information:**

The online version contains supplementary material available at 10.1186/s12931-022-02105-7.

## Background

Idiopathic pulmonary fibrosis (IPF) is a diffuse parenchymal lung disease with an incidence of approximately 2.8- 9.3 cases per 100,000 individuals per year [1]. Current concepts suggest repetitive alveolar epithelial micro-injuries followed by the accumulation and proliferation of fibroblasts and myofibroblasts in the lung parenchyma to contribute to IPF pathology. As part of the ensuing lung tissue remodeling, increased deposition of extracellular matrix (ECM) components such as collagen and elastin by myofibroblasts and other cell types triggers uncontrolled lung tissue scarring with subsequent loss of lung function [2]. Histopathologic indicators of the usual interstitial pneumonia (UIP) pattern of lung fibrosis include the so-called ‘honeycombing’-like lung tissue remodeling typically developing in the subpleural regions of the lung, as well as focal structures of subepithelial fibroblast/myofibroblast proliferation along with ECM protein deposition known as fibroblast foci [3]. Overall, prognosis of IPF is poor with an estimated median survival of 3–4 years after diagnosis [4].

Matrix metalloproteinases (MMPs) are zinc-dependent endopeptidases and are secreted as inactive zymogens, thus requiring further activation via their calcium-binding domain [5]. Pro-MMP-2 is activated by forming ternary complexes with MT1-MMP (MMP14) and TIMP-2 on the cell surface leading to degradation of collagen type I and III [6, 7]. MMPs are capable of degrading nearly all kinds of proteins of the ECM. At the same time, MMPs are also involved in the processing of signaling proteins such as cytokines and chemokines, thereby modulating their release and/or activity [8, 9]. For example, MMP-2 and MMP-9 are able to release active pro-fibrotic TGF-β1 by proteolytic cleavage of latency-associated peptide (LAP) bound to TGF-β1 [10, 11]. Because of their ECM-degrading activities, MMPs are considered to contribute to regulation of lung regeneration and repair in response to lung injury, and dysregulated MMP activation may be part of the imbalance between ECM deposition versus degradation characterizing many interstitial lung diseases (ILD) [12].

MMP-2 and MMP-9 (also known as gelatinases A and B) particularly degrade gelatin, elastin, as well as various types of collagens. Both proteases are expressed by alveolar epithelial cells, fibroblasts and fibrocytes, and have been detected in BAL fluids and lungs of IPF patients [13–19]. However, the role of MMP-2 and MMP-9 in pulmonary fibrogenesis is still understudied, likely due to the fact that deletion of MMP-2 (gelatinase A) may be functionally compensated by MMP-9 (gelatinase B), making assessment of a direct contribution of one single gelatinase in lung fibrogenesis rather difficult. Therefore, in the current study, we have examined the role of single MMP-2 or MMP-9 deletion and of combined MMP-2/-9 double deletion in a well-defined murine model of lung fibrosis induced by adenoviral exposure to biologically active TGF-β1.

## Materials and methods

### Animals

MMP-2 KO mice (B6.129P2-MMP-2 < tm1Ito > /ItoRbrc) were purchased from RIKEN BioResource Center (Tsukuba, Ibaraki, Japan) [20] and MMP-9 KO mice (B6.FVB(Cg)-MMP-9 < tm1Tvu > /J) were purchased from The Jackson Laboratory (Bar Harbor, Main, USA) [21]. A (B6.129P2-Mmp2tm1Ito/ItoRbrc × B6.FVB(Cg)-Mmp9tm1Tvu/J) F2 generation was created and genotyped to obtain offsprings deficient for both, MMP-2 and MMP-9. Mice homozygous for both mutants were inbred to generate the strain B6.Cg-Mmp2tm1ItoMmp9tm1Tvu/. DNA was isolated from ear punches using Snooplex© Fast Prep Kit (GvG, Leipzig) and used for PCR (50 ng/µL DNA). Mice were genotyped by PCR using allele-specific oligonucleotides. Three oligonucleotides for MMP-2 specific PCR (Common Rev: 5′-CCGGGACAGGAACGTACTGGGTTC-3′, For: 5′-GTGCTACTGCAGGATAAACTGA-TG-3′, Mut: 5′-GCGCCTACCGGTGGATGTGGAATGTGTGCG-3′) yielded a 794 bp amplicon for the homozygous wt/wt genotype, a 310 bp amplicon for the homozygous tm/tm genotype and 794 bp + 310 bp amplicons for the heterozygous wt/tm genotype. Three primers for MMP-9 specific PCR (wt Rev: 5′-TCCCACTTGAGGCCTTTGA-3′, Common For: 5′-TCCTCCATCCACAGGCATAC-3′, olMR6218 tm Rev: 5′-CCTTCTATCGCCTTCTTGACG-3′) yielded a 223 bp amplicon for the homozygous wt/wt genotype, a 400 bp amplicon for the homozygous tm/tm genotype and 223 bp + 400 bp amplicons for the heterozygous wt/tm genotype (See Additional file 1: Fig. S1). All the mice (single and double mutant mice as well as WT siblings) were bred under specific pathogen-free (SPF) conditions at the Central Animal Facility of Hannover Medical School. Female and male mice were used in experiments at the age of 10–20 weeks. All procedures were in accordance with the German Animal Welfare Legislation and approved by the local Institutional Animal Care and Research Advisory Committee and permitted by the Lower Saxony State Office for Consumer Protection and Food Safety (reference number 16/2249).

### Treatment of mice

Mice were anaesthetized by intraperitoneal (i.p.) injection of 75 mg/kg body weight (b.w.) ketamine hydrochloride (Anesketin, Albrecht, Aulendorf, Germany) and 3 mg/kg b.w. xylazine hydrochloride (Rompun, Bayer, Leverkusen, Germany). An Abbocath-T catheter (26G; Hospira Venisystems, Lake Forest, USA) was inserted under visual control into the trachea of mice. Adenoviral vector carrying the cDNA for biologically active porcine TGF-β1 (AdTGF-β1) or empty control vector (AdCL) were prepared as previously described [22] and were applied orotracheally (o.t.) at 1 × 10^8^ plaque-forming units (PFU) in 50 µl PBS per mouse. Mice were then brought back to their cages with free access to food and water.

### Sampling of explant lung tissue from patients with interstitial lung disease and disease controls for determination of MMP-2 and MMP-9 levels

Lung tissue samples were collected from upper lung central (Ulc) and upper lung peripheral (Ulp) as well as lower lung central (Llc) and lower lung peripheral (Llp) locations of freshly explanted lungs from patients with ILD (mostly IPF) by an experienced pathologist (D.J.). All ILD patients presented a fully developed usual interstitial pneumonia (UIP) pattern of lung fibrosis. Characteristics of patients and disease controls (patients with lung carcinoma) are shown in Additional file 1: Table S1. Tissue specimens were handled anonymously according to the principles expressed in the Declaration of Helsinki. The study was designed and performed following the requirements of the local ethics committee at MHH (ethics vote no. 2702-2015).

For determination of MMP-2 and MMP-9 levels, lung samples were weighed and homogenized in PBS supplemented with protease inhibitor cocktail (Merck, Darmstadt, Germany) to a final concentration of 500 mg tissue/ml PBS. MMP-2 and MMP-9 levels were measured in lung homogenate supernatants of ILD patients and disease controls using commercially available ELISA kits (R&D Systems, Minneapolis, US). MMP-2 and MMP-9 levels measured in lung homogenates of ILD and disease control patients are expressed as mean values of lung tissue specimen collected per patient.

### Bronchoalveolar lavage

Bronchoalveolar lavage of mice was performed as previously described [23–25].

### Western blot

Western blot analysis of MMP-2 and MMP-9 in lung and BAL samples was performed as described [24]. MMP-2 and MMP-9 protein was detected using monoclonal anti-MMP-2 (Cell Signaling, Frankfurt am Main, Germany) and anti-MMP-9 (Abcam, Cambridge, US) antibodies diluted 1:1,000 in 1% milk and anti-beta-actin antibodies were diluted 1:25,000 in 1% milk. Beta actin (Sigma-Aldrich, Steinheim, Germany) served as loading control. Specificity of employed antibodies was confirmed by mass spectrometry at the Proteomics Core Facility of Hannover Medical School (data not shown).

### ELISA

Quantification of MMP-2, MMP-9 and TGF-β1 in BAL fluids was performed using ELISA according to the manufacturer’s instructions (R&D Systems, Wiesbaden, Germany).

### Gelatin zymography of MMP-2 and MMP-9 activity in BAL fluids of mice

We determined enzymatic activities of MMP-2 and MMP-9 in BAL fluids of mice either exposed to control vector or AdTGF-β1 for 21 days. Briefly, BAL proteins were concentrated using Amicon Ultracentrifugal filters with a cutoff size of 10 kDa, according to the manufacturer’s protocol (Merck, Darmstadt, Germany). Next, protein contents of individual samples were determined using Pierce-Micro BCA protein assay kits (Thermo Scientific, Waltham, US), and equal amounts of total protein per sample were then mixed with equal volumes of 4 × non-reducing Laemmli buffer (BioRad, Feldkirchen, Germany). A total of 10 µg protein per sample was loaded per lane, and electrophoresis was performed using 7.5% SDS-PAGE gels containing 1% gelatin (Sigma Aldrich, Steinheim, Germany) in the separation gel. After electrophoresis, gels were then transferred to washing buffer (2.5% Triton X-100; 50 mM Tris–HCL pH 7.5; 5 mM CaCl_2_; 1 µM ZnCl_2_) and were shaken at a low speed for 2 × 30 min to elute SDS. Subsequently, gels were placed in incubation buffer (1% Triton X-100; 50 mM Tris–HCl pH 7.5; 5 mM CaCl_2_; 1 μM ZnCl_2_) followed by incubation at 37 °C for 24 h. Afterwards, gels were stained with Coomassie brilliant blue (G250, Biorad, Feldkirchen, Germany) for 30 min with slow shaking and were then decolorized in destaining solution until transparent bands due to MMP-2 and MMP-9 enzymatic digestion of gelatin contents became visible against the blue (undigested gelatin) background. Finally, gels were subjected to visualization of MMP-2 and MMP-9 enzyme activities using a gel image analysis system (Vilber Lourmat, Ebhardzell, Germany).

### Hydroxyproline assay

Hydroxyproline dye binding assay for determination of lung collagen contents was performed as previously described [24–26].

### Lung histopathology

WT and MMP-2/-9 dKO mice either exposed to AdCL or AdTGF-β1 were euthanized on day 21 with an overdose of isoflurane (Baxter, Unterschleissheim, Germany), and lungs were subsequently inflated with PBS-buffered formaldehyde solution (4%, Roth, Karlsruhe, Germany). Lungs were removed and immersed in formaldehyde solution for 24 h at room temperature. Paraffin-embedded lung tissue section (3 μm) were stained with hematoxylin/eosin (HE) and analyzed using an Olympus BX-53 microscope at × 10 original magnification.

### Assessment of lung function

Lung function of mice was assessed as recently described [27]. Briefly, mice were anaesthetized as described above, and were then tracheotomized and cannulated with a shortened 20-gauge needle that was firmly fixed to the trachea. Mice were connected to a FlexiVent respirator (SCIREQ, Montreal, Canada) and were invasively ventilated (tidal volume 10 ml/kg b.w., respiratory rate 150/min, positive endexpiratory pressure (PEEP) 3 cm H_2_O), as described elsewhere [27, 28]. Then, two recruitment maneuvers (deep inflation of the lung, 30 cm H_2_O) were applied following three pressure-controlled quasi-static pressure–volume (PV) loops. Forced oscillation technique (FOT), involving baseline ventilation for 5 min with 30 s intervals of low frequency perturbations was conducted. Inspiratory capacity (IC) was calculated during deep inflation, and tissue elastance (H) was calculated by fitting the constant-phase model to impedance spectra obtained during FOT. Quasi-static compliance (Cst) was de termined by the mean of the three PV loop values using Salazar-Knowles equation [27, 28].

### Statistical analysis

Statistical significance between groups was assumed when p values were < 0.05. Differences between experimental groups were analyzed by Mann–Whitney *U* test using GraphPad Prism software (version 8.0).

## Results

### Determination of MMP-2 and MMP-9 levels in lung tissue of ILD patients and disease controls

**Fig. 1:**
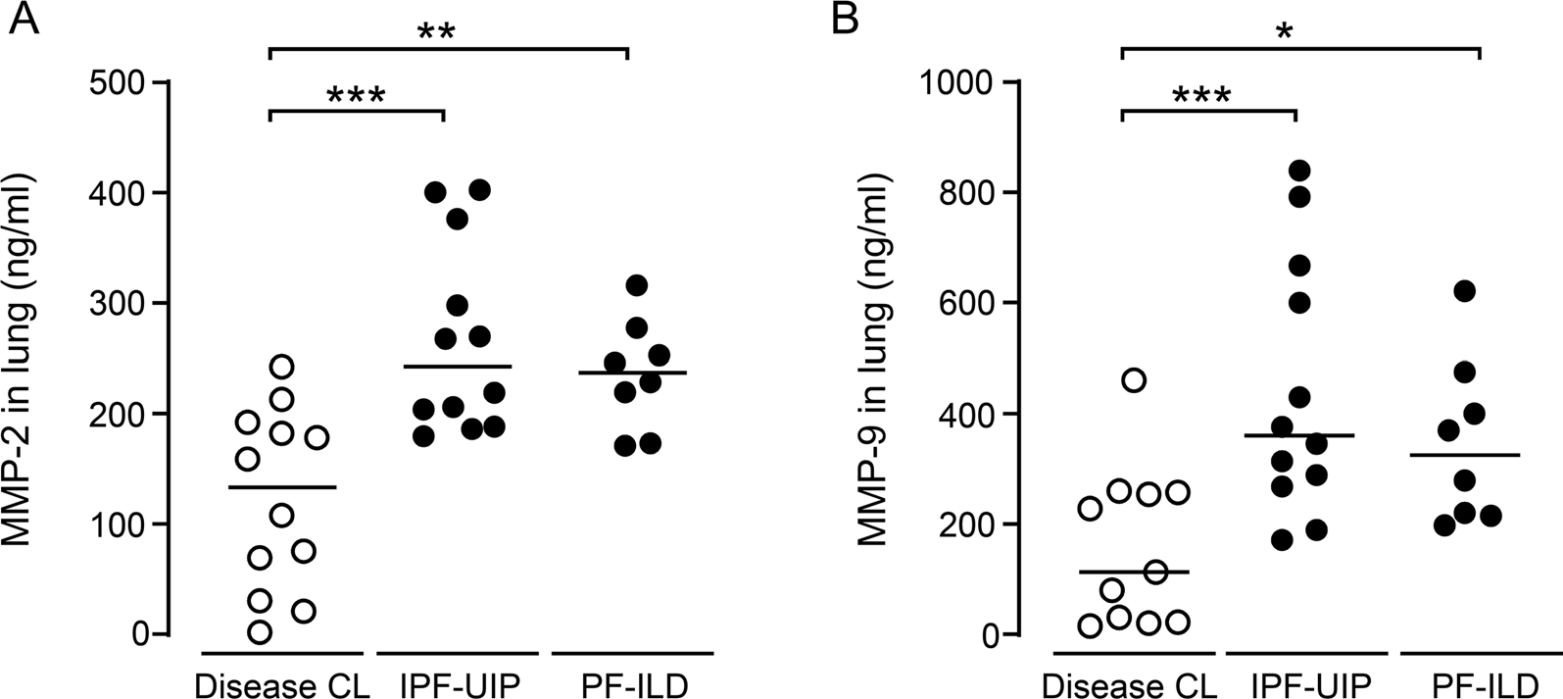
Determination of MMP-2 and MMP-9 levels in lung tissue of ILD patients and disease controls. **A**, **B** MMP-2 (**A**) and MMP-9 (**B**) protein levels in explant lung tissue samples from lower and upper lung aspects of patients with IPF-UIP and non-IPF-UIP pattern, and from tumor-free lung tissue of disease control patients. Data are presented as scatter plots with median values indicated as horizontal lines of n = 10–12 patients per group. *p < 0.05, ***p < 0.001 compared to disease control (Mann–Whitney U test)

In initial experiments, we determined MMP-2 and MMP-9 levels in explant lung tissue from patients with ILD relative to disease controls (for patient characteristics see Additional file 1: Table S1). As shown in Fig. 1, we found significantly increased levels of both MMP-2 and MMP-9 protein in lung homogenates with no significant differences between patients with IPF-UIP or non-IPF-UIP.

### Analysis of MMP-2 and MMP-9 protein levels in experimental lung fibrosis in mice

**Fig. 2:**
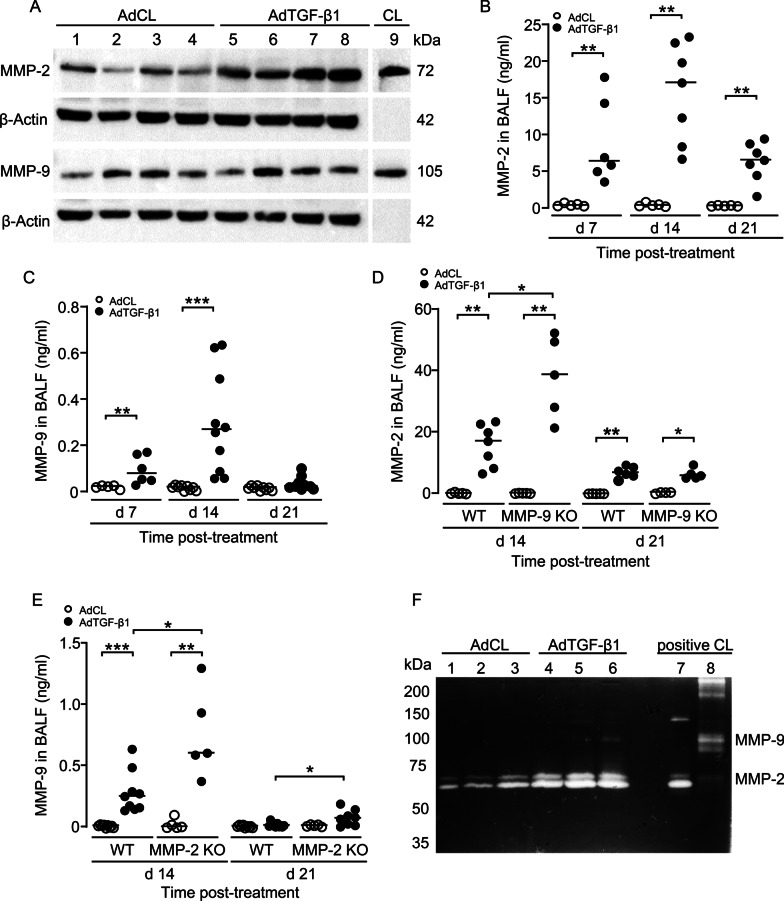
Analysis of MMP-2 and MMP-9 protein levels in experimental lung fibrosis in mice. **A** Western blot analysis of MMP-2 (72 kDa) and MMP-9 (105 kDa) proteins in lungs of AdCL versus AdTGF-β1-treated mice, as indicated. Beta-Actin (42 kDa) was used as a loading control. **B**, **C** MMP-2 (**B**) and MMP-9 (**C**) protein levels were measured in BALF of mice treated with AdCL (white dots) or AdTGF-β1 (black dots) at 7, 14 and 21 days post-treatment, as indicated. **D** MMP-2 protein levels in AdCL (white dots) or AdTGF-β1 (black dots) exposed WT and MMP-9 KO mice at days 14 and 21 post-treatment, as indicated. **E** MMP-9 protein levels in AdCL (white dots) or AdTGF-β1 (black dots) exposed WT and MMP-2 KO mice at days 14 and 21 post-treatment, as indicated. **F** Gelatin zymography of latent MMP-2 (72 kDa), or active MMP-2 (64 kDa), and latent MMP-9 (105 kDa) in BAL fluids of AdCL versus AdTGF-β1 treated mice at 21 days post-treatment, as indicated. Data in (B-E) are shown as scatter plots with median values indicated as horizontal lines of n = 5–10 mice per time point and treatment group. *p < 0.05, **p < 0.01, ***p < 0.001 (Mann–Whitney U test)

We next analyzed MMP-2 and MMP-9 protein expression in lung tissue lysates of AdCL and AdTGF-β1 exposed WT mice (day 21 post-treatment) by western blotting. As shown in Fig. 2A, we observed low MMP-2 and -9 protein expression in AdCL-treated mice, but strongly increased MMP-2 protein levels in lungs of WT mice upon AdTGF-β1 treatment, whereas MMP-9 showed a much weaker expression.

We then quantified MMP-2 and MMP-9 protein levels in BAL fluids of AdCL versus AdTGF-β1 exposed WT mice. As shown in Fig. 2B and C, MMP-2 and -9 protein levels were nearly undetectable in BAL fluids of AdCL exposed mice, but significantly increased in BALF of AdTGF-β1-exposed mice at day 7 post-treatment, with peak levels observed by day 14 and a strong decline by day 21 (Fig. 2B, C). Overall, MMP-2 protein levels were approximately 50-fold higher than MMP-9 protein levels by day 14 post-AdTGF-β1 (Fig. 2B, C).

We then measured protein levels of MMP-2 in MMP-9 KO mice and vice versa MMP-9 protein levels in MMP-2 KO mice either treated with control vector or AdTGF- β1. As illustrated in Fig. 2D and E, MMP-2 levels were significantly increased in BALF of MMP-9 KO mice relative to MMP-2 levels observed in AdTGF-β1 treated WT mice. Similarly, MMP-9 protein release in BALF of MMP-2 KO mice was significantly increased when compared to AdTGF-β1 treated WT mice (Fig. 2E).

Since MMP-2 and MMP-9 are released as latent proteins requiring further processing to achieve full enzymatic activities, we finally determined enzymatic activities of MMP-2 and MMP-9 in BALF of mice with established fibrosis using gelatin zymography assays. As shown in Fig. 2F, we found low MMP-2 specific gelatinolytic activities in BALF of AdCL-treated mice, which substantially increased in BALF of AdTGF-β1 treated mice. However, we were not able to detect MMP-9-specific gelatinolytic activity in AdCL-treated mice, and only a weakly detectable MMP-9 enzyme activity in BALF of AdTGF-β1 treated mice (Fig. 2F). Together, the current data demonstrate that (1) both proteases MMP-2 and MMP-9 are released in lungs of AdTGF-β1 exposed mice with MMP-2 > > MMP-9, (2) deletion of one gelatinase triggered a compensatory upregulation of the other gelatinase in lungs of AdTGF-β1 mice, and (3) MMP-2 rather than MMP-9 was found to be the primary enzymatically active gelatinase in lungs of AdTGF-β1 exposed mice.

### MMP-2 and/or MMP-9 knockout does not affect experimental lung fibrosis in mice

**Fig. 3:**
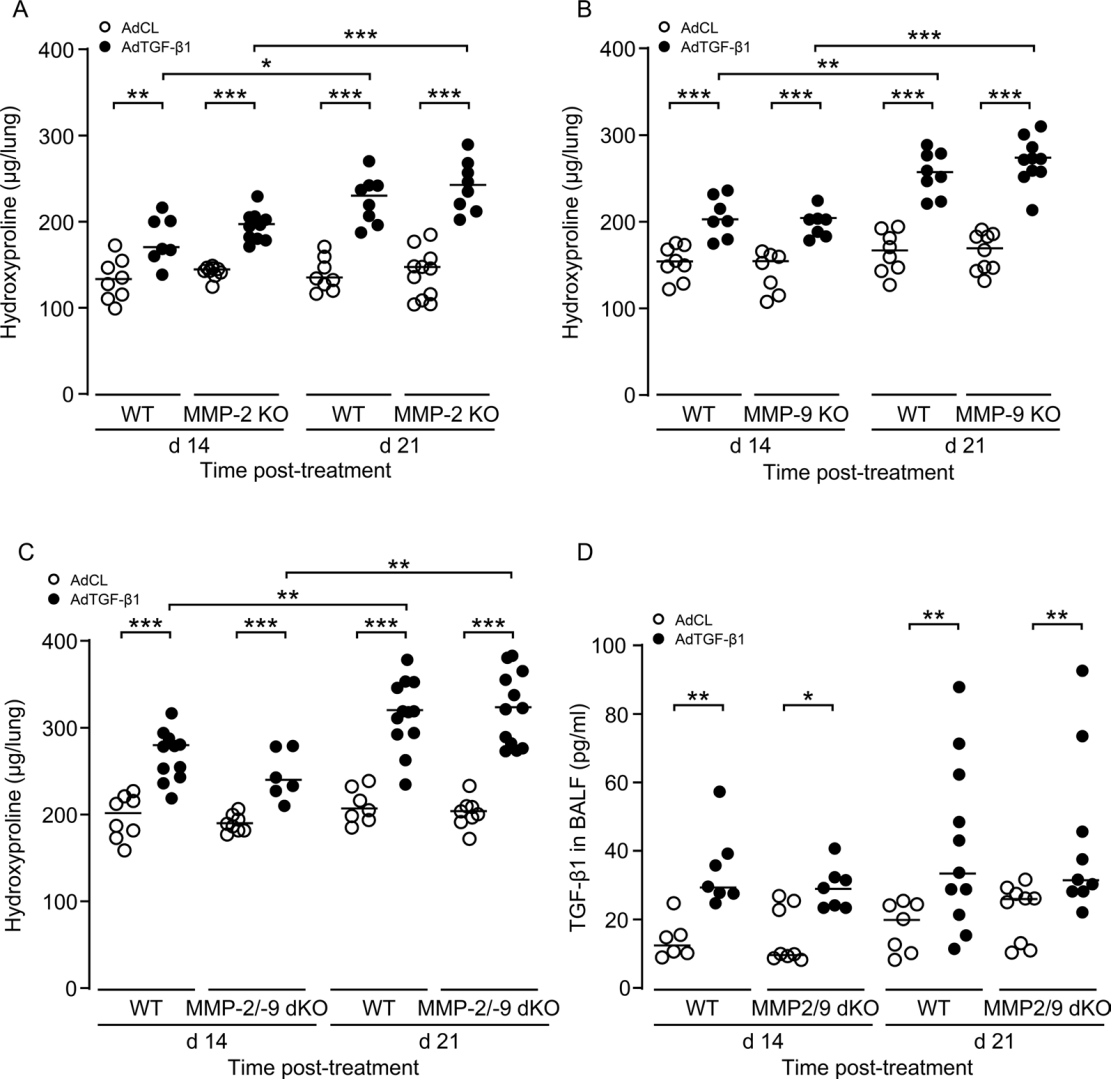
MMP-2 and/or MMP-9 knockout does not affect experimental lung fibrosis in mice. **A**–**C** Lung collagen contents were measured in WT, MMP-2 KO mice (**A**), or MMP-9 KO mice (**B**), or MMP-2/-9 dKO mice (**C**) either treated with empty AdCL vector (white dots) or AdTGF-β1 (black dots) at day 21 post-treatment. **D** TGF-β1 protein levels in AdCL (white dots) or AdTGF-β1 (black dots) exposed WT and MMP-2/-9 dKO mice at days 14 and 21 post-treatment, as indicated. Data are shown as scatter plots with median values indicated as horizontal lines of n = 6–11 mice per time point and treatment group, and are representative of two independent experiments. *p < 0.05, **p < 0.01, ***p < 0.001 (Mann–Whitney U test)

Based on the observed strong induction of MMP-2 and -9 in both human lung explant tissue of ILD patients and mice with established fibrosis, we determined what effect single MMP-2 or MMP-9 knockout would have on AdTGF-β1 induced lung fibrosis in mice. As shown in Fig. 3A and B, we found significantly increased lung collagen contents in all experimental groups (WT, MMP-2, MMP-9) on day 14 and 21 post-AdTGF-β1, relative to control vector treatment, with no significant difference between groups. Of note, no differences in lung collagen contents were observed between male and female WT or MMP2/9 dKO mice after exposure to AdCL or AdTGF-β1 (data not shown).

Since lack of gelatinase A/MMP-2 may be functionally compensated by gelatinase B/MMP-9, we next examined hydroxyproline contents in lungs of mice deficient of both MMP-2 and MMP-9. Again, AdTGF-β1 treated WT and MMP-2/-9 double KO mice exhibited significantly increased hydroxyproline contents on day 14 post-treatment, which further increased by day 21, with no significant difference between experimental groups (Fig. 3C). Since many of the MMPs, including MMP-2 and MMP-9, are able to activate latent TGF-β in vitro we examine TGF-β1 levels in BALF of AdTGF-β1 treated mice. We found uniform TGF-β1 levels in WT and MMP-2/-9 dKO mice on day 14 and 21 post-treatment (Fig. 3D). These data show that either lack of single MMP-2 or MMP-9 or combined MMP-2/-9 double knockout did not exert major effects on lung collagen deposition in our experimental rodent model of lung fibrosis.

### Lung histopathology of WT and MMP-2/-9 dKO mice with AdTGF-β1-induced lung fibrosis

**Fig. 4:**
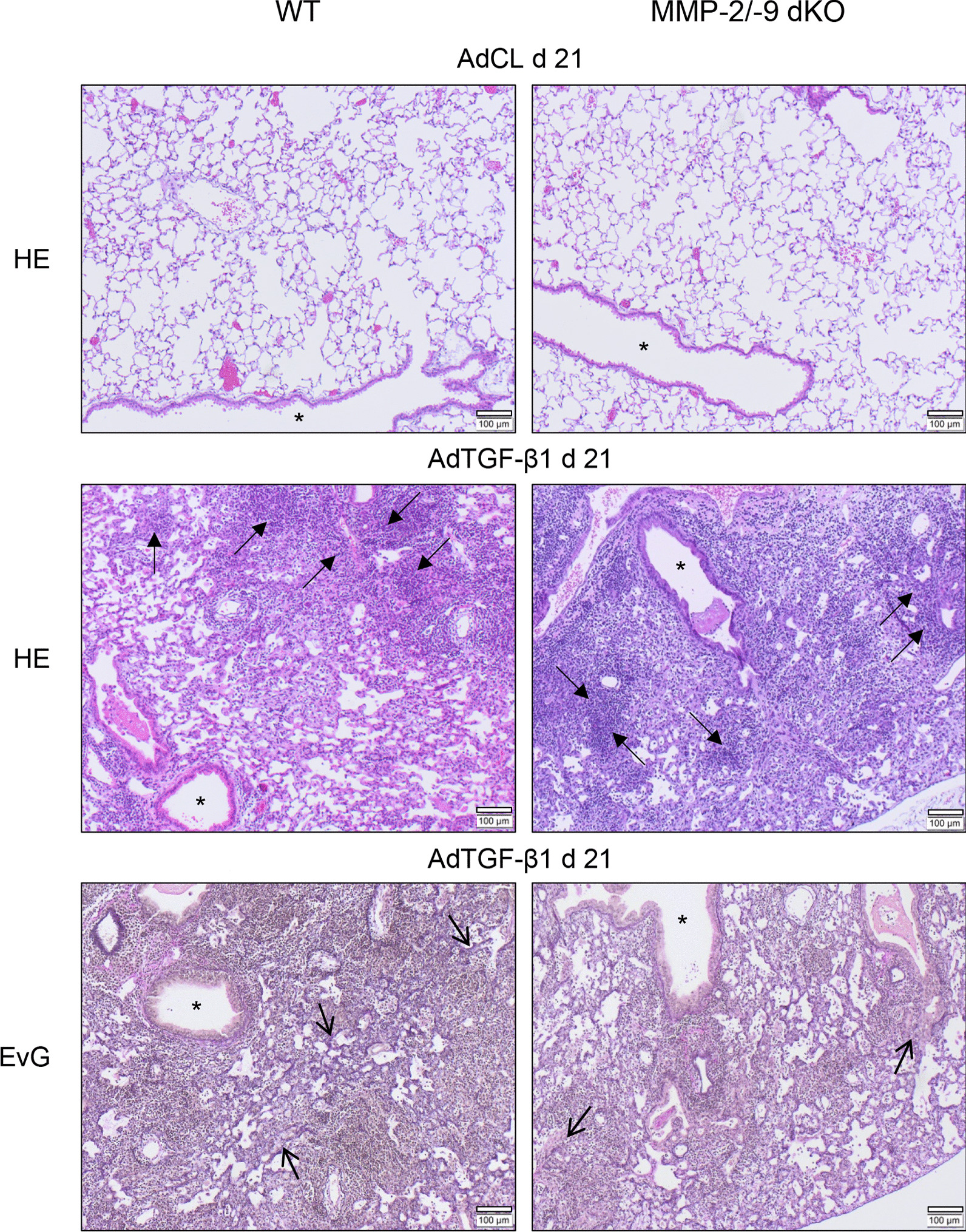
Lung histopathology of WT and MMP-2/-9 dKO mice with AdTGF-β1-induced lung fibrosis. WT and MMP-2/-9 dKO mice were exposed to AdCL vector or AdTGF-β1 for 21 days. Subsequently, lung sections were stained with hematoxylin/eosin or Elastica van Gieson (EvG) for assessment of lung tissue remodeling. The provided histology is representative of n = 4 mice per experimental group. Closed arrows, lymphoplasmacellular infiltrates; open arrows, areas of increased fibrotic remodeling; asterisk, bronchus (original magnification, × 10; scale bar, 100 µm)

Histopathological inspection of lung tissue showed a normal lung architecture with unaffected bronchi and bronchioli in AdCL-exposed WT and MMP-2/-9 dKO mice. As expected, we found substantial lung tissue remodeling in WT mice in response to AdTGF-β1, which was characterized by peribronchovascular and partly interstitial accentuated lymphocytic infiltrates, along with widened alveolar septa and interstitial collagen deposition (Fig. 4). Similar lung histology was also noted in MMP-2/-9 dKO mice, with no major differences in lung tissue remodeling between groups (Fig. 4).

### MMP-2/-9 double knockout does not worsen lung function in experimental lung fibrosis in mice

**Fig. 5:**
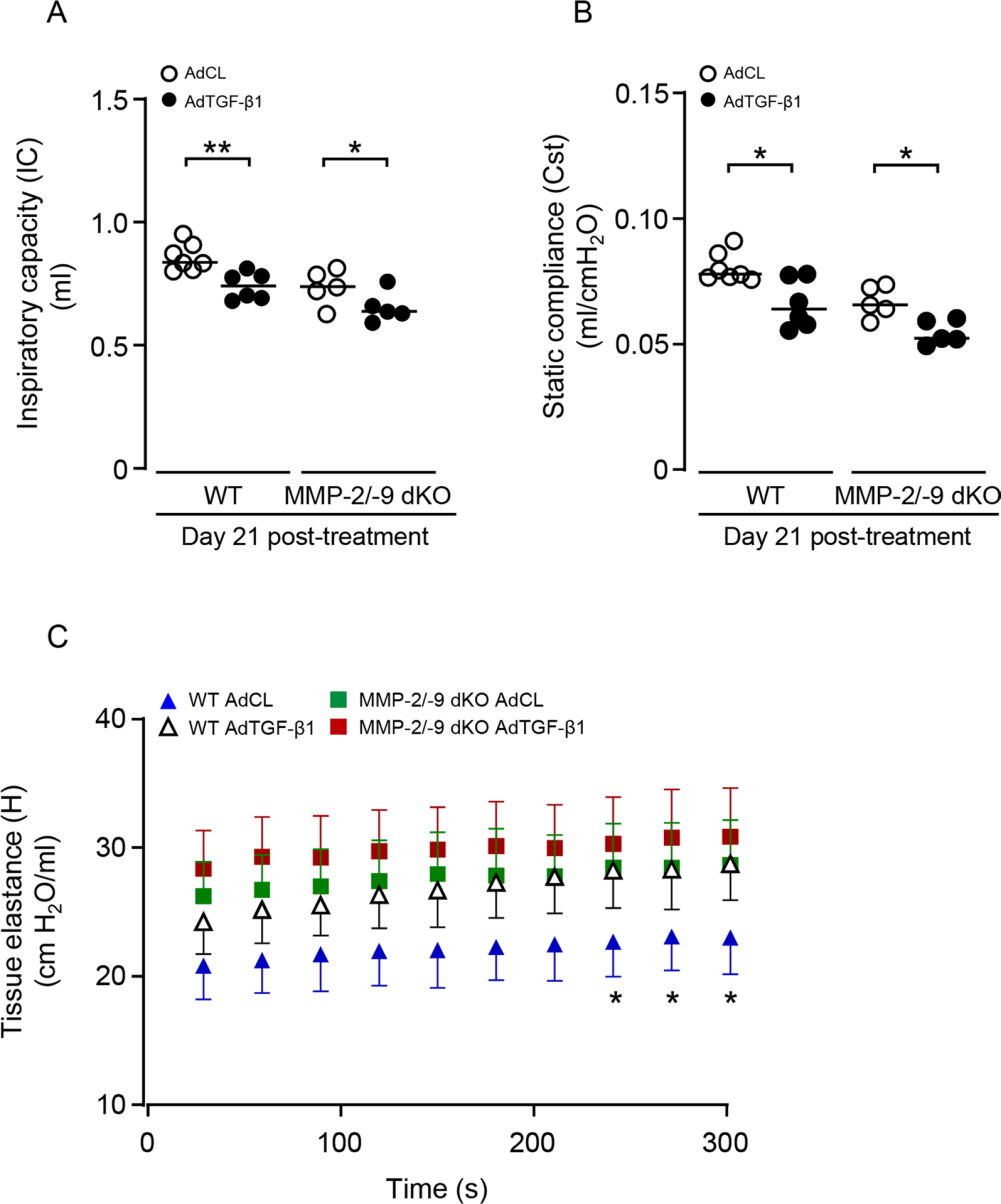
MMP-2/-9 double knockout does not worsen lung function in experimental lung fibrosis in mice. WT and MMP-2/-9 dKO mice were treated with empty control vector (AdCL) or AdTGF-β1 for 21 days and were then subjected to invasive lung function testing. **A**, **B** Inspiratory capacity (IC; **A**) and static compliance (Cst, **B**) in the lungs of AdCL treated (white dots) or AdTGF-β1 (black dots) treated WT or MMP-2/-9 dKO mice, as indicated. **C** Tissue elastance (H) analysed on day 21 after AdCL (blue triangle) or AdTGF-β1 (white triangle) treatment of WT mice, and AdCL (green squares) or AdTGF-β1 (red squares) treatment of MMP-2/-9 dKO mice. *indicates p < 0.05, **p < 0.01 of AdTGF-β1 relative to AdCL exposed WT mice. Data are shown as scatter plots with median values indicated as horizontal lines (**A**, **B**) or mean ± SD (**C**) of n = 5–8 mice per time point and treatment group and are representative of two independent experiments. *p < 0.05, **p < 0.01 (**A**, **B**) (Mann–Whitney U test), or two-way ANOVA (C)

Based on the finding that both WT mice and MMP-2/-9 dKO mice showed comparably increased lung hydroxyproline levels and lung tissue remodeling in response to AdTGF-β1, in the next set of experiments, we questioned whether MMP-2/-9 dKO would impact lung function in these mice, relative to WT controls. As shown in Fig. 5, both WT and MMP-2/-9 dKO mice demonstrated a reduced inspiratory capacity (IC) upon AdTGF-β1 treatment, relative to AdCL mice, with no significant differences between groups (Fig. 5A). Similarly, both WT and MMP-2/-9 dKO mice developed increased lung tissue elastance (H) upon AdTGF-β1 treatment, which was more pronounced in AdTGF relative to AdCL treated WT mice, while reaching significance at later time points of the pressure/volume curve. However, tissue elastance was not significantly different between AdTGF-treated WT and MMP-2/-9 dKO mice (d 21; Fig. 5C). Consistent with this finding, lung quasi-static compliance (Cst) reflecting elastic recoil of lung tissue was similarly decreased in WT and MMP-2/-9 dKO mice, again with no significant difference between groups (Fig. 5B).

## Discussion

Excess deposition of ECM proteins in the lung interstitium is a hallmark of lung fibrosis and critically contributes to lung stiffness, shortness of breath, and eventually respiratory failure. Due to their potential to remove ECM, matrix metalloproteinases have gained substantial attention in the context of IPF pathogenesis [8, 9, 12]. Here, we made a first contribution to evaluate the roles of MMP-2 and MMP-9 in a well-defined model of lung fibrosis using single and double KO mice lacking MMP-2 and -9, thereby establishing experimental conditions where lack of one gelatinase cannot be compensated by the other gelatinase. Based on biochemical along with histopathological examinations and invasive lung function tests in mice, we suggest that MMP-2 and MMP-9 play only a subordinate role in lung fibrosis in mice.

Previous studies showed that particularly MMP-1, -2, -3, -9 and -13 were upregulated in IPF patients and experimental models of lung fibrosis [9, 16–18, 29, 30]. We also found elevated levels of MMP-2 and MMP-9 protein in lung tissue of both patients with IPF and non-IPF lung fibrosis with UIP pattern, consistent with previous reports [16–19, 31, 32]. Similarly, MMP-2 and MMP-9 protein levels were also increased in BALF and lung homogenates of mice with AdTGF-β1-induced fibrosis. Other reports using the bleomycin model also showed higher MMP-2 and MMP-9 protein levels in rats and mice [33–35]. More specifically, immunohistochemical studies showed a prevailing extracellular activity for MMP-2, while MMP-9 was primarily active intracellularly in bleomycin-challenged rodents [36]. Adding to these data, we show that MMP-2 was released in AdTGF-β1 treated mice more abundantly than MMP-9, and found it to be the primarily active among these two gelatinases by gelatin zymography assays. Expectedly, knockout of one gelatinase led to compensatory increase of the other gelatinase in BALF of AdTGF-β1 treated mice. In sum, we show that i) MMP-2 was more abundantly expressed in AdTGF-β1 exposed mice, and ii) showed a stronger gelatinolytic activity compared to MMP-9, and iii) was strongly upregulated in lungs of AdTGF-β1 exposed MMP-9 KO mice. While these data suggest that MMP-2 is the primarily active gelatinase during lung fibrogenesis, the data also imply that a reasonable study of gelatinase A or B in lung fibrosis should be done in MMP-2/-9 double KO but not in single-mutant mice.

Several studies showed that MMP-2 is particularly expressed and released by bronchial and alveolar epithelial cells and fibroblasts, whereas MMP-9 is mainly expressed by leukocyte subsets, including alveolar macrophages, neutrophils, and lymphocytes as well as club cells [13–16, 37, 38]. MMP-2 deficient mice demonstrated upregulation of MMP-9 protein in lymphocytes and lung endothelial cells, but not dermal fibroblasts [39].

In addition to their undisputed roles as ECM degrading enzymes, more recent studies have linked MMPs to fibrogenesis via regulation of inflammatory mediators, other proteases, growth factors and posttranslational processing of chemokines [40, 41]. As an example, latency-associated protein (LAP) is covalently bound to latent TGF-β binding protein (LTBP), and MMP-2 and -9 is able to release latent LAP/LTBP complex resulting in increases of the active form of TGF-β1, thereby promoting lung fibrosis progression [10, 11]. Here, we used replication-deficient adenoviral vectors to transfer the cDNA of biologically active porcine TGF-β1 to induce lung fibrosis in mice. In this model, the vector-derived active TGF-β1 typically vanishes by day 10 post adenoviral delivery, and progression of fibrosis is achieved by subsequently released endogenous TGF-β1, as shown previously [26]. Therefore, since the TGF-β1 protein observed on days 14 and 21 post-treatment comprises only endogenously released murine TGF-β1, we expected less lung fibrosis particularly in the double mutant mice, which however was not observed. Based on these data, the roles of gelatinases A and B in experimental rodent models of lung fibrosis appear to be rather limited.

MMP-2 and MMP-9 have been attributed rather discordant roles in lung fibrosis. For example, a previous study did not find differences in hydroxyproline levels between MMP-9 KO and WT mice in bleomycin-induced fibrosis [42]. In contrast, overexpression of MMP-9 in alveolar macrophages of transgenic mice decreased fibrotic lesions and hydroxyproline levels after bleomycin challenge in another study [13]. The role of MMP-2 in lung fibrosis has not been studied in MMP-2 KO mice so far. Some preliminary data in human MMP-2 expressing mice suggested less lung fibrosis and inflammatory cells upon bleomycin challenge [43]. In contrast, in experimental liver fibrosis, MMP-2 KO mice responded with increased fibrotic remodeling [44, 45], while inhibition of MMP-2 attenuated experimental kidney fibrosis [46, 47]. To the best of our knowledge, our study is the first to characterize lack of effect of MMP-2 and MMP-2/-9 deletion in experimental lung fibrosis in mice. Of note, MMP-2/-9 dKO mice were studied in non-fibrosis related conditions like cancer, cerebral ischemia or antibody-induced arthritis [48–50]. In this context, MMP-2/-9 dKO mice showed a delayed wound healing compared to WT mice [48] and were protected from hemorrhagic transformation during early cerebral ischemia [49]. In a model of antibody-induced arthritis, MMP-2 KO mice showed a severe phenotype, while MMP-9 KO mice showed a milder form of arthritis, while MMP-2/-9 dKO mice showed no difference in arthritis compared to WT mice [50].

In summary, we evaluated for the first time the roles of MMP-2 and MMP-9 in murine lung fibrosis evoked by adenoviral delivery of biologically active TGF-β1, using both MMP-2 and MMP-9 KO mice as well as MMP-2/-9 double KO mice. In light of the currently presented biochemical assays, lung histology and lung function assays, we did not find any major contribution of MMP-2 or -9 to lung fibrogenesis in mice.

## Conclusion

These data strongly support the view that the two gelatinases MMP-2 and MMP-9 play a subordinate role in lung fibrogenesis in the described model of lung fibrosis.

## Supplementary Information

**Additional file 1: Figure S1.** Genotyping of MMP-2 KO, MMP-9 KO, and MMP-2/-9 double KO mice. Schematic overview of genotyping of MMP-2 KO and MMP-9 KO mice and genotyping of MMP-2 KO, MMP-9 KO, and MMP-2/-9 double KO mice by PCR. **Table S1.** Patient characteristics. Characteristics of ILD-patients and disease controls.

## Data Availability

The datasets generated during and/or analysed during the current study are available from the corresponding author on reasonable request.

## Abbreviations

IPF: Idiopathic pulmonary fibrosis
ECM: Extracellular matrix
MMP-2: Matrix metalloproteinase 2
MMP-9: Matrix metalloproteinase 9
WT: Wild-type
KO: Knockout
dKO: Double knockout
ELISA: Enzyme-linked immunosorbent assay
BALF: Bronchoalveolar lavage fluid
TGF-β1: Transforming growth factor beta 1
UIP: Usual interstitial pneumonia
ILD: Interstitial lung fibrosis
