# Supplementary material for: Role of matrix metalloprotease-2 and MMP-9 in experimental lung fibrosis in mice
Source: Respir Res. 2022 Jul 8;23:180. doi: 10.1186/s12931-022-02105-7 (PMC9270768; doi:10.1186/s12931-022-02105-7)
Supplement: Supplementary file 1 — Additional file 1: Figure S1. Genotyping of MMP-2 KO, MMP-9 KO, and MMP-2/-9 double KO mice. Schematic overview of genotyping of MMP-2 KO and MMP-9 KO mice and genotyping of MMP-2 KO, MMP-9 KO, and MMP-2/-9 double KO mice by PCR. Table S1. Patient characteristics. Characteristics of ILD-patients and disease controls. [file 12931_2022_2105_MOESM1_ESM.pdf]

Additional File 1

Role of Matrix Metalloprotease-2 and MMP-9 in experimental lung fibrosis in mice

Tina Bormann, Regina Maus, Jennifer Stolper, Meritxell Tort Tarrés, Christina Brandenberger, Dirk Wedekind, Danny Jonigk, Tobias Welte, Jack Gauldie, Martin Kolb, and Ulrich A. Maus

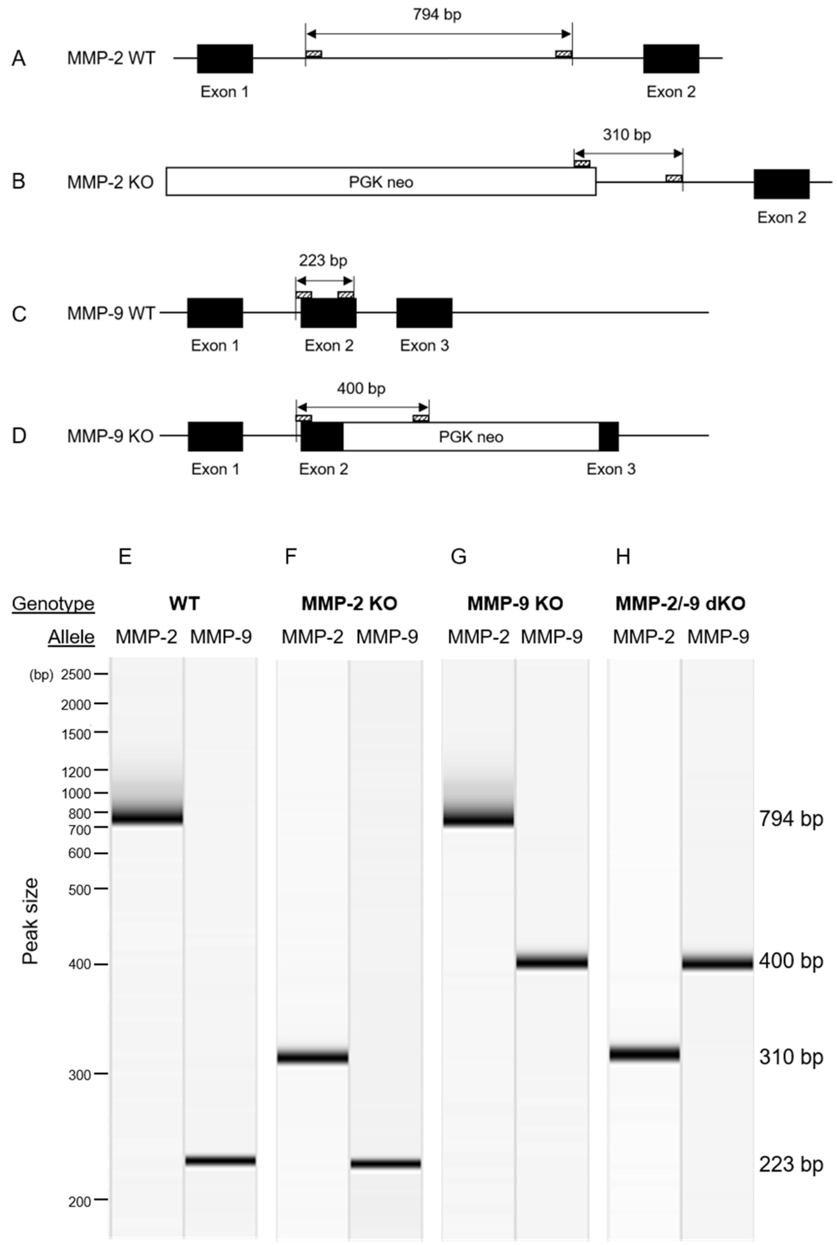

Figure S1. Genotyping of MMP-2 KO, MMP-9 KO, and MMP-2/-9 double KO mice

(A-D) Schematic overview of genotyping of MMP-2 KO (A,B) and MMP-9 KO mice (C,D) with specific primer binding sites for PCR amplification. (E-G) Genomic DNA was extracted from ear punches of WT, MMP-2 KO, MMP-9 KO and MMP-2/-9 dKO mice, and after MMP-2 and MMP-9 specific PCR amplifications, DNA was subjected to advanced capillary electrophoresis using the QIAxel system (Qiagen, Hilden, Germany). (E) PCR products of WT MMP-2 (794 bp) and WT MMP-9 (223 bp) alleles. (F) Genotype of MMP-2 KO mice displaying the expected WT MMP-9 allele and the mutated MMP-2 allele (310 bp). (G) Genotype of MMP-9 KO mice displaying the expected WT MMP-2 allele and the mutated MMP-9 allele (400 bp). (H) Genotypic profile of MMP-2/-9 dKO mice exhibiting mutated alleles for both MMP-2 and MMP-9.

Table S1 Patient characteristics

Characteristics	Lung fibrosis	Disease control
Total number	20	12
Subgroup	IPF (12), PF-ILD (8), including HP (5)[1]	Neoplasia
Men/women	11/9	6/6
Age (range)	57 (31-65)	66 (46-76)
FEV1 (% predicted)	41.5±11.0	79.7±15.6
FVC (% predicted)	40.9±11.8	89.6±19.8
TLC (% predicted)	50.7±9.9	96.6±15.8

Values are expressed as mean±SD, and age as median (range). FEV, forced expiratory volume; FVC, forced vital capacity; HP, hypersensitivity pneumonitis; IPF, idiopathic pulmonary fibrosis; PF-ILD, progressive fibrosing interstitial lung disease; TLC, total lung capacity; UIP, usual interstitial pneumonia. [1]PF-ILD with UIP pattern including hypersensitivity pneumonitis.
